# Do the Benefits of Male Circumcision Outweigh the Risks? A Critique of the Proposed CDC Guidelines

**DOI:** 10.3389/fped.2015.00018

**Published:** 2015-03-18

**Authors:** Brian D. Earp

**Affiliations:** ^1^Uehiro Centre for Practical Ethics, University of Oxford, Oxford, UK

**Keywords:** circumcision, Centers for Disease Control and Prevention, sexually transmitted diseases, HIV, autonomy, medical ethics, benefit vs. risk, female genital mutilation

## Abstract

The Centers for Disease Control and Prevention (CDC) have announced a set of provisional guidelines concerning male circumcision, in which they suggest that the benefits of the surgery outweigh the risks. I offer a critique of the CDC position. Among other concerns, I suggest that the CDC relies more heavily than is warranted on studies from Sub-Saharan Africa that neither translate well to North American populations nor to circumcisions performed before an age of sexual debut; that it employs an inadequate conception of risk in its benefit vs. risk analysis; that it fails to consider the anatomy and functions of the penile prepuce (i.e., the part of the penis that is removed by circumcision); that it underestimates the adverse consequences associated with circumcision by focusing on short-term surgical complications rather than long-term harms; that it portrays both the risks and benefits of circumcision in a misleading manner, thereby undermining the possibility of obtaining informed consent; that it evinces a superficial and selective analysis of the literature on sexual outcomes associated with circumcision; and that it gives less attention than is desirable to ethical issues surrounding autonomy and bodily integrity. I conclude that circumcision before an age of consent is not an appropriate health-promotion strategy.

## Introduction

The Centers for Disease Control and Prevention (CDC) have announced a set of provisional guidelines concerning male circumcision, in which they suggest that the benefits of the surgery outweigh the risks [(1), p. 2]. Although their main focus is on the potential for male circumcision to provide partial protection against female-to-male, heterosexually transmitted HIV, due to the comparatively rare occurrence of such infections in the United States, the CDC notes in its draft report that the “the overall public health benefit [to] the entire U.S. population may be limited” [(2), np]. Nevertheless, the proposed CDC guidelines have generated significant interest among public health professionals, as well as among the population at large. In this brief report, I highlight a few of the key scientific and ethical issues worth considering in interpreting these new recommendations.

## Following the AAP

First, the CDC appears largely to be following the American Academy of Pediatrics (AAP), whose 2012 policy statement and technical report have already been subjected to numerous international critiques (3–12)^1^. While these critiques are not necessarily definitive, it is worth noting that the CDC authors do not actually engage with them in their scientific discussion. Thus, they fail adequately to address the concerns that have been raised in these previous writings about the manner in which the AAP – and by extension, the CDC – conducted its analysis of the available literature on male circumcision, and presented its findings to the public^2^.

Among other issues, critics have pointed out that the bulk of the data used to justify the AAP/CDC policies was derived from studies of adult circumcision carried out in sub-Saharan Africa – a geographic region whose epidemiological environments and patterns of disease transmission are dissimilar, along numerous dimensions, to those elsewhere in the world (13–16). This is important, because the spread of disease, including sexually transmitted infections, is determined much more by socio-behavioral and situational factors than by strictly anatomical-biological factors, such as the presence or absence of a foreskin (17, 18). In other words, the apparent findings from these studies cannot be simply mapped on to non-analogous public health environments (15), nor to circumcisions performed earlier in life, i.e., before an age of sexual debut (19). As Bossio et al. (20) argue in a recent comprehensive review, not referenced by the CDC, “At present … the majority of the literature on circumcision is based on research that is not necessarily applicable to North American populations” (p. 2847).

The CDC acknowledges this ‘translation’ problem: “Much of the data related to HIV and STI prevention are from randomized clinical trials (RCTs) conducted among men in sub-Saharan Africa in regions with high rates of heterosexually acquired HIV infection. In the United States [by contrast], the prevalence of HIV and lifetime risk of HIV infection are generally much lower than [in] sub-Saharan Africa. Also, most new HIV infections in the United States are attributed to male–male sex, a population for whom male circumcision has not been proven to reduce the risk of HIV acquisition” [(1), p. 1].

## Benefit vs. Risk

In addition to such empirical limitations, the proposed CDC guidelines exhibit conceptual and ethical limitations as well. Conceptually, the CDC relies on an inappropriate construal of risk in its benefit vs. risk analysis, since it appears to interpret “risk” as referring (primarily or exclusively) to the “risk of surgical complications.” To begin with, the actual incidence of surgical complications is not known, due to the poor quality of the available data on this question as well as conflicting definitions of (and ways of measuring) “complications” (21). Thus, as Garber (5) has noted, “it is inconceivable that the AAP [and by extension, the CDC] could have objectively concluded that the benefits of the procedure outweigh the risks when the ‘true incidence of complications’ isn’t known” (p. 69). Nevertheless, it has been argued that the CDC working group underestimated even the known risks of circumcision, by focusing on the comparatively rare, immediate surgical risks and complications that occur soon after the operation, while ignoring or downplaying the comparatively common intermediate and long-term complications, such as meatal stenosis, which may require a surgical correction (22).

What if it could be shown, however, that the benefits of circumcision did in fact outweigh the (overall) risk of surgical complications? Even so, the CDC test would still be ill-conceived. This is because the standard heuristic for evaluating non-therapeutic surgery (i.e., surgery performed in the absence of disease or deformity) is not benefit vs. “risk of surgical complications” but rather benefit vs. risk of harm (23, 24). In this case, at least one relevant harm would be the inherent loss of a healthy, functional, and erotogenic penile structure (25, 26), amounting to approximately 30–50 cm^2^ of densely innervated, elastic genital tissue in the adult organ (12, 27). Since this tissue can be manipulated during sex and foreplay, resulting in a range of concomitant sense perceptions (28), and since it protects the sensitive head of the penis from abrasion as well as from drying out over time (25), its surgical removal entails a number of arguably adverse outcomes, even if the circumcision is properly performed (19).

To its discredit – and contrary to the practice of most non-US-based national health organizations (10) – the CDC nowhere in its proposed guidelines mentions, much less explores in any detail, the actual anatomy or functions of the penile foreskin (22). As Fleiss and Hodges (29) ask, “How can parents make a rational decision about circumcision when they are told nothing about the part that will be cut off?” (p. xii). For a point of comparison, imagine a report by the CDC discussing the health benefits of prophylactic mastectomy, in which the only implied harms of the procedure were “surgical complications,” and in which the anatomy and functions of the breasts were nowhere described.

## Harm

Most basically, the CDC’s approach runs counter to the conventional bioethical (and legal) view that unnecessary surgeries, and especially those that remove non-diseased, functional tissue from an individual without his consent, are in and of themselves harmful. As a California Appeals Court recently held [quoted in Ref. (30)], “[I]t seems self-evident that unnecessary surgery is injurious and causes harm to a patient. Even if a surgery is executed flawlessly, if the surgery were unnecessary, the surgery in and of itself constitutes harm” (p. 469).

The only other potential harm that the CDC appears to have entertained is the possibility of diminished sexual experience, finding that: “Adult men who undergo circumcision generally report minimal or no change in sexual satisfaction or function” [(1), p. 7]. However, the CDC’s appraisal of the literature on this point is as superficial as it is selective^3^. As Bossio et al. (20) noted in their recent review: “Adverse self-reported outcomes associated with foreskin removal in adulthood include impaired erectile functioning, orgasm difficulties, decreased masturbatory functioning (loss in pleasure and increase in difficulty), an increase in penile pain, a loss of penile sensitivity with age, and lower subjective ratings of penile sensitivity” (p. 2853, internal references omitted). While “other studies have found no significant differences in self-reported sexual functioning following adult circumcision” (*ibid*.), it must be remembered that a lack of statistical significance does not entail a lack of underlying effect (31). For example, in one of the studies cited by the CDC, “several questions were too vague to capture possible differences between circumcised and not-yet circumcised participants [such that] non-differential misclassification of sexual [outcomes] probably favored the null hypothesis of no difference, whether an association was truly present or not” [(32), p. 313].

Finally, as noted earlier, the CDC ignores the fact that any sensation in the foreskin itself is necessarily eliminated by circumcision, as are any sexually relevant (e.g., masturbatory) functions that require its manipulation. As I have argued elsewhere: “To say that circumcision has ‘little or no effect’ on sexual experience is to adopt an extremely narrow conception of that term” [(19), p. 44]. More generally, studies of adult male circumcision often lack adequate long-term follow-up, and assess only a limited range of sexual outcome variables (19, 20).

## Risk and Risk Perception

In addition to its inadequate conception of risk, the CDC portrays the risks that it does consider in a potentially misleading manner. This is because it describes the “benefits of male circumcision [in terms of] relative-risk reductions (e.g., a 50% reduction from a 2% risk of an STI to a 1% risk), whereas any associated harm is expressed as an absolute risk (e.g., a 2–4% risk of adverse events)” [(1), p. 2]. In other words, the purported benefits of circumcision are described in figures whose values may be quite large despite being derived from small absolute percentages [see, e.g., Ref. (12)], whereas the potential harms are described in ‘small’ numbers (i.e., percentages expressing absolute risk). This may have the effect of inflating the perceived likelihood and/or magnitude of the potential benefits of circumcision, and – by contrast – deflating the perceived drawbacks and harms, especially in the minds of those who are unversed in interpreting medical statistics [see, e.g., Ref. (33, 34)]. Since this is likely to include the very individuals whom the CDC suggests should undergo “counseling” about male circumcision, such differential risk-description poses a threat to the ethical validity of obtaining their informed consent [see generally, Ref. (35–37)]^4^.

This is not a trivial concern. As Hoffmann and Del Mar (38) have shown, patients – in general – already tend to overestimate the benefits of proposed medical interventions, and already tend to underestimate the harms. Thus, unless patients (or parents) clearly understand that their (or their child’s) absolute risk of, e.g., heterosexually acquired HIV infection in the United States is very low – indeed zero before an age of sexual debut – the relative-risk reduction figures presented by the CDC could give the wrong impression.

## Health Benefits? Taking into Account Gender and Ethics

On the question of health benefits, suppose it could be shown that removing the labia majora of infant girls reduced their risk of acquiring a urinary tract infection (since there would be fewer folds of moist genital tissue in which bacteria could find a home), as well as, say, cancers of the vulva – or even HIV (39). It is not biologically implausible. In fact, in countries in which female ‘circumcision’ is culturally normative, it is often said to confer a range of such benefits, including “a lower risk of vaginal cancer … less nervous anxiety, fewer infections from microbes gathering under the hood of the clitoris, and protection against herpes and genital ulcers” [(40), p. 258]. In addition, female ‘circumcision’ in such countries is often described as ‘more hygienic’ as well as more esthetically pleasing (41).

Now, it is not usually recognized that female ‘circumcision’ falls on a spectrum; that some forms of it are less invasive than male circumcision (including several forms that do not involve modification of the clitoris); and that it is sometimes done for reasons other than (attempted) control of sexuality (42–47). Nevertheless, it is actually illegal in Western countries to conduct the very research by which such ‘health benefits’ could be ‘discovered’ in the first place. This is because non-therapeutic surgeries performed on the genitals of healthy girls – no matter how slight, nor under what material conditions – are deemed to be impermissible mutilations in Western law (45).

Presumably, this is due to concerns about respect for sexual self-determination, a desire to protect children’s (future) autonomy [see Ref.(48–50)], and a recognition of their basic moral and legal rights to bodily integrity and to security of the person (51–53). Moreover, since there are more effective, but much less invasive, ways of preventing and/or treating most of the diseases to which the external female genitalia may sometimes fall prey – such as the use of soap and water for simple hygiene, the adoption of safe sex practices, and the administration of antibiotics, if required – it seems reasonable to argue that pre-emptive surgery toward the same ends would fail the test of proportionality (54).

Taken together, these considerations suggest that little girls should be free to grow up with their genitals intact, and to decide, at an age of understanding, whether they would like to undergo permanent alterations to their ‘private parts,’ and if so, for what reasons (and what kind). The same considerations apply equally to boys [(40, 46, 50, 55–57); see also Ref. (58, 59) for further discussion].

## Timing

With respect to timing, the CDC (1) states, “Neonatal male circumcision is, safer, [*sic*]^5^ and heals more rapidly than circumcision performed on older boys [and] men, and is less expensive” (p. 4). There are two points to consider here. First, as Svoboda and Van Howe (60) have argued: “complications may certainly be better *documented* for adults, who have the knowledge and wherewithal to complain if something goes wrong; but there is no consistent evidence that properly performed adult circumcision is actually riskier.”^6^ Second, “It is true that it can be more costly, but only if proper pain control is used: general anesthesia is contra-indicated in infants, meaning that the surgery is performed either with no pain control or with sub-optimal pain control, driving down costs at the expense of humane treatment” [(60), np; see also Ref. (61, 62)]^7^.

A further concern – again – has to do with the interpretation of risk. According to even proponents of circumcision, such as Brian Morris (63), the absolute likelihood of clinically significant, non-resolvable surgical complications associated with the surgery is low, regardless of the age at which it is performed. Thus, even if one were to grant that there is a relative-risk reduction in the incidence of adverse events – over and above the loss of erogenous tissue – this consideration would not be morally decisive.

To see why this is the case, consider the hypothesis that any number of surgeries might be (statistically) ‘safer’ if carried out in the neonatal period. The prior question, however, is whether the surgery itself is ethically sound. For example, imagine that it could be shown that removing a child’s earlobes for non-therapeutic reasons was ‘less risky’ if it were done to an infant. Nevertheless, from a moral perspective, such an intervention would be seen as clearly impermissible (52, 64).

To its credit, the CDC (65) seems to recognize such concerns, although it does not discuss them in any detail in its actual (proposed) recommendations. Instead, in a less accessible background technical report, it states that: “Delaying male circumcision until adolescence or adulthood obviates concerns about violation of autonomy” (p. 39), and therefore any “disadvantages associated with [such a deferral] would be ethically compensated to some extent by the respect for the [bodily] integrity and autonomy of the individual” (pp. 39–40).

## Conclusion

As Murphy (66) has argued: “Biomedical research and its social applications are almost always worthy of sustained critical scrutiny” (p. 11). In the case of circumcision, in particular – originally a ritual practice with a long history of being dubiously medicalized (67) – it is important to be especially skeptical (12). At the end of the day, it is not altogether clear that a minor reduction in the absolute risk of certain infections or diseases – whose prevalence in developed nations is generally low, and whose occurrence can typically be avoided by other, less injurious means^8^ – is worth the ‘trade-off’ of losing a part of one’s penis. What is certain, however, is that the answer to this question is likely to be highly subjective, and to depend upon numerous, unpredictable, and ultimately personal factors [see Ref. (24)]. Therefore, it should be up to the affected individual himself (or indeed herself, in analogous circumstances) to decide about permanent genital-modification surgeries at such a time as he or she can meaningfully factor in his or her own preferences and values [see Ref. (46, 48, 51)]^9^. Circumcision before an age of consent is not a desirable health-promotion strategy, given more effective, and less ethically problematic, alternatives.

## Conflict of Interest Statement

The author declares that the research was conducted in the absence of any commercial or financial relationships that could be construed as a potential conflict of interest.

## Footnotes

^1^Note that replies and counter-replies to some of these critiques have been published. For example, in response to an accusation of cultural bias by 38 senior physicians and “representatives of general medical associations and societies for pediatrics, pediatric surgery, and pediatric urology” from England, Canada, and Northern Europe [(3), p. 796], the AAP argued that it is Europeans, not Americans, who are culturally biased – only against circumcision rather than in favor of it (68). Offering a different perspective, I have suggested elsewhere that: “By [implying that] a cultural norm favoring the non-therapeutic, non-consensual surgical modification of a child’s penis is somehow on par with, or just as reasonable as, a medical-ethical norm favoring the *avoidance* of such surgery unless it is absolutely required, the AAP committee simply reveals its cultural hand” [(69); for further discussion, see Ref. (12)].

^2^See Earp (70), for a discussion of the proper role of scientific organizations in shaping the public’s understanding of medicine.

^3^In the 61-page technical report which forms the basis for its proposed recommendations, the CDC (65) dedicates a total of four sentences to the possible effects of circumcision on sexual sensation, function, and/or satisfaction. Moreover, it fails to reference, much less discuss, several well-known studies providing evidence of negative effects of circumcision on sexuality [e.g., Ref. (71–74)], including some that were available within the period of review. Instead, the CDC relies on a small assortment of apparently randomly selected articles from the literature purporting to show a lack of adverse outcomes, without citing any of the published critiques of those studies. By contrast, a comprehensive, critical, and nuanced discussion of the available evidence concerning the various sexual (and other) effects of circumcision can be seen in the recent work of Bossio et al. (20). As these authors note, a previous “systematic review” by Morris and Krieger (75), the first of whom is a long-time advocate of neonatal circumcision (12, 76), is “not a meta-analysis, thus, no statistical analyses of the data have been performed; instead, the article presents the authors’ interpretation of trends.” Problematically, however, “Morris and Krieger do not report the results of this review collapsed across study quality. The conclusion they draw – that circumcision has no impact on sexual functioning, sensitivity, or sexual satisfaction – does not necessarily line up with the information presented in their review, which is mixed” [(20), p. 2854].

^4^Related to this worry, it is important that patients (or parents) not mistake the CDC’s recommendation about *counseling* regarding circumcision – a process whose conclusion for any individual patient may very well be that it should not be done – for a recommendation about circumcision generally, i.e., that it should be performed as a routine procedure. But as patients (or parents) might reasonably ask, “Why would the CDC both recommend that I be routinely counseled about the benefits and risks of circumcision, as well as strongly suggest that the former outweigh the latter, unless it ‘wanted’ me (or my son) to be circumcised?” Such mixed messaging further reduces the possibility of obtaining (truly) informed consent, whether from the patient or from his putative proxy.

^5^It should be noted that the CDC background report is replete with typographical errors such as this one, inaccurate referencing, and other signs of careless scholarship. For example, Sandra Hassink – a CDC supporter and President of the AAP – notes that the CDC literature review is out of date, that the CDC confuses adverse events with remedies for adverse events, and that “some paragraphs are not well organized or include non-sequiturs” [(77), np].

^6^Emphasis added. As they go on to state: “Only three studies have directly compared the complication rates of infant and later circumcision. One found no difference; another found a significantly greater rate following infant circumcision; and a third found the opposite when using a Plastibell device” (np, internal refs omitted). The issue of direct comparison is important. This is because the claim that infant circumcision has fewer risks compared to later (i.e., adult) circumcision is often grounded in appeals to retrospective comparisons of different studies (or data sets), conducted by different researchers, with results drawn from discordant populations. Thus they do not adequately control for such variables as the skill of the practitioner, the specific device(s) used, the sterility of the environment, and so on. For an in-depth discussion, see one of the formal peer reviews of the CDC background materials (78).

^7^As Bellieni et al. [(62), p. 1] note, “no procedure has been found to definitively eliminate pain; the gold standard procedure to make MC totally painfree has not yet been established.”

^8^As Kluge (82) asks, “Is it ethically appropriate to perform circumcisions because there is some statistical evidence that a potentially curable disease with a low incidence rate may be prevented by surgery, even though the disease also occurs in people who have undergone the surgery and the incidence rate of the disease in countries where the surgery is not routinely performed is similar to that in countries where it is? If the answer to this question is Yes, then the same underlying principle should be applied to all similar cases: whenever there is statistical evidence that a potentially curable disease or condition with a low incidence rate could be prevented by surgery, but the evidence also indicates that the incidence rate is the same (or similar) in other countries where the surgery is not routinely performed, we should still perform the surgery in every person in whom the disease or condition might develop. All sorts of medical conditions would be implicated. I suspect that we would be operating nonstop on just about every part of the human body if we took this stance. I shudder to think of the cost – and the implications for public health” (p. 1452).

^9^To elaborate: by suggesting that the benefits of circumcision *outweigh* the risks (as opposed to merely presenting what is known about the potential benefits and risks, such as their type, their likelihood under various conditions, etc., without presuming to know how these factors should be balanced against one another), the CDC makes a number of value-laden [see Ref. (79)], and, as some would argue, potentially culturally biased [see Ref. (3)], ethical assumptions [see Ref. (80), p. 30]. For example, the CDC assumes that the foreskin itself has little or no value, which is a relatively uncommon view outside of circumcising cultures; it assumes, as I have argued, that the (unknown) risk of surgical complications is the only, or at least the main, morally relevant cost to circumcision, etc. But given the contentious nature of non-therapeutic circumcision, from both a moral and a scientific perspective [see Ref. (81)], as well wide variance, both culturally and individually, with respect to norms about intact vs. modified genitals, it seems reasonable to argue that it is not the CDC that should be making such idiosyncratic ethical valuations [see Ref. (70)], but rather the individual who will be affected by the surgery [see Ref. (19, 24, 46)].
